# Pathological characteristics of lung tumors in Sri Lanka 2017–2021

**DOI:** 10.1111/1759-7714.15206

**Published:** 2024-01-07

**Authors:** Yasith Mathangasinghe, Sameera Wijayawardhana, Udeshika Perera, Ramani Punchihewa, Saman Pradeep

**Affiliations:** ^1^ Department of Anatomy Genetics and Biomedical Informatics, Faculty of Medicine University of Colombo erted Colombo Sri Lanka; ^2^ Department of Anatomy and Developmental Biology, School of Biomedical Sciences, Faculty of Medicine, Nursing and Health Sciences Monash University Clayton Victoria Australia; ^3^ Department of Anatomy, Faculty of Medicine University of Kelaniya Kelaniya Sri Lanka; ^4^ Cambridge University Hospital, Addenbrooke's Hospital Cambridge UK; ^5^ Department of Pathology National Hospital for Respiratory Diseases Welisara Sri Lanka; ^6^ Department of Thoracic Surgery National Hospital for Respiratory Diseases Welisara Sri Lanka; ^7^ Royal Papworth Hospital Cambridge Biomedical Campus Cambridge UK

**Keywords:** adenocarcinoma, demographics, epidemiology, lung neoplasms, pathology

## Abstract

The prevalence of lung cancer is steadily increasing globally, and it is projected to become the second most prevalent cancer in men by 2030. Lung cancer is the leading cause of cancer‐related deaths worldwide, accounting for approximately 3.61% of total fatalities. Despite its significant impact, many Asian countries, including Sri Lanka, lack precise data on the epidemiological patterns of lung tumors. This study pioneers a comprehensive exploration in Sri Lanka, delving into the demographic and clinicopathological characteristics of lung cancer patients. The study included 733 consecutive patients with lung tumors from 2017 to 2021, with a median age of 59 years. The most common site of tumors was the right lower lobe and left upper lobes. Adenocarcinoma was the most prevalent histopathological type of primary malignant lung tumors, while colorectal adenocarcinomas were the most common cause of metastatic deposits in the lungs. The most common benign tumor was hamartoma. Significantly, our findings unveiled associations between patient demographics and tumor types, underscoring the importance of factoring in age and gender in diagnostic assessments. Notably, the absence of a dedicated lung cancer screening program in Sri Lanka underscores the critical reliance on clinical suspicion and accurate diagnostic methods.

## Graphical abstract

This is the first comprehensive analysis of lung tumors in Sri Lanka and one of Asia's largest, featuring detailed histopathological analysis and demographic correlations. Adenocarcinomas were prevalent among primary malignant tumors, with colorectal adenocarcinomas leading in metastatic deposits. Crucially, our findings revealed correlations between patient demographics and tumor types, emphasizing the pivotal role of age and gender in diagnostic assessments.

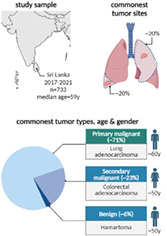

The prevalence of lung cancer is steadily increasing, and it is projected to become the second most prevalent cancer in men by 2030.^1^ Globally, both the incidence and mortality rates of lung cancer have experienced an exponential rise.^2^ Lung cancer ranks as the leading cause of cancer‐related deaths worldwide and in Sri Lanka, comprising approximately 3.61% and 1.82% of total fatalities, respectively.^3^ However, the precise figures regarding lung tumors in Sri Lanka remain uncertain. In 2017, for the first time, we reported the pathological analysis of lung tumors in Sri Lanka,^4^ and now we report the outcomes of a 5 year follow‐up of our study.

We conducted a descriptive study at the National Hospital for Respiratory Diseases (NHRD) of Sri Lanka, spanning from 2017 to 2021. NHRD stands as the exclusive tertiary care center dedicated to respiratory diseases in the country, offering specialized diagnosis and treatment. Notably, it serves as the primary referral institution for lung tumor patients in Sri Lanka, enabling us to assemble a nationally representative patient sample.^5^ We included all patients who had their lung tumors analyzed through histopathological examination. We analyzed the most recent biopsy for patients who underwent serial biopsies to avoid duplicating results. A single consultant histopathologist examined the specimens using standard histological and immunohistochemical stains. We defined patients with multiple metastases as those exhibiting at least one positive biopsy result indicating malignant cytology or histology of the lung, along with radiological evidence of multiple metastases. We categorized lung tumors based on the World Health Organization 2021 classification.^6^ Our investigation focused on examining associations between demographic characteristics and pathological characteristics of lung tumors.

The study included individuals aged 1–89 years, with a median age of 59 (q1 = 49, q3 = 66) years. Out of the 733 patients, 445 (60.7%) were males. The right lower lobe and left upper lobes were the most common site of tumors (*n* = 145, 19.8% each). Benign neoplasms were present in 42/733 cases (5.7%), while the most common benign lung tumor was hamartoma (*n* = 29/42, 69.0%). Among 524 primary malignant lung tumors, adenocarcinoma was the most prevalent (*n* = 285/524, 54.4%). Out of 167 metastatic deposits in the lungs, the majority were due to colorectal adenocarcinomas (27/167, 16.2%) (Table 1).

**TABLE 1:** Distribution of histopathological types of lung tumors in the study population.

Type of tumor		Frequency (*n*) and percentage (%) of tumors (*n* = 733)		Gender				Age (years)		
				Male		Female		Median	q1	q3
		*n*	%	*n*	%	*n*	%			
Benign	Total	42	5.7%	26	62%	16	38%	51	42	62
	Pulmonary hamartoma	29	4.0%	19	66%	10	34%	52	43	65
	Benign spindle cell tumor	9	1.2%	5	56%	4	44%	50	30	61
	Adenoma	4	0.5%	2	50%	2	50%	50	30	55
Primary malignant	Total	524	71.5%	345	66%	179	34%	61	53	67
	Adenocarcinoma	285	38.9%	175	61%	110	39%	62	54	68
	Squamous cell carcinoma	123	16.8%	109	89%	14	11%	63	58	69
	Neuroendocrine tumors	74	10.0%	37	50%	37	50%	53	40	60
	Sarcoma	20	2.7%	10	50%	10	50%	49	30	59
	Salivary gland‐type tumors	5	0.7%	2	60%	3	17%	44	33	57
	Non‐small cell lung carcinoma, NOS	8	1.1%	7	88%	1	12%	62	58	68
	Lymphoma	4	0.5%	2	50%	2	50%	51	31	69
	Adenosquamous carcinoma	3	0.4%	2	67%	1	33%	67	53	67
	Large cell carcinoma	2	0.3%	1	50%	1	50%	58	‐	‐
Metastasis	Total	167	22.8%	74	44%	93	56%	51	35	60

Abbreviation: NOS, not otherwise specified.

The distribution of age among different types (i.e., benign, primary malignant, secondary) of masses was found to have significant differences, according to an independent samples Kruskal–Wallis test (*W* = 70.029, *p* < 0.001). Post hoc comparisons indicated that the patients with primary malignant tumors had a higher median age (61 years) compared to those with benign (51 years) and secondary malignant (51 years) lung tumors. Pearson's *χ* ^2^‐test showed significant associations between sex and the type of tumor (*χ* ^2^(df = 2, *n* = 733) = 24.633, *p* < 0.001). Post‐hoc tests with Bonferroni correction showed that the benign and primary malignant lung tumors were more common among males, while secondary malignant tumors were more common in females.

To date, Sri Lanka, along with several other developing countries in South Asia, lacks a screening program for lung malignancies. Consequently, a high degree of clinical suspicion and accurate radiological and histopathological evaluations are necessary to achieve better outcomes. We found that primary lung malignancies occurred at a higher rate (71.5%) compared to metastatic neoplasms (22.8%). This difference may be attributed to the National Cancer Institute's primary focus on patients with advanced metastatic neoplasms, resulting in fewer referrals to the NHRD. Adenocarcinoma was the most commonly reported metastatic lung tumor worldwide,^7^ which aligns with our findings. Specifically, colorectal adenocarcinoma and soft tissue sarcoma were the most prevalent extra‐pulmonary malignant tumors among metastatic lung malignancies. Regarding primary lung malignancies, both our study and cancer registries in the USA identified adenocarcinoma as the most frequent histopathological type, while squamous cell carcinoma ranked second.^7^ The prevalence of hamartoma as the most common benign lung tumor in our study corresponds with Western statistics.^8^ Smoking, a major modifiable risk factor for lung cancer worldwide, has significant implications for disease prevalence and mortality.^2^, ^9^ Recent trends in smoking are reflected in lung cancer rates,^2^, ^9^ with a higher prevalence among men than women in Sri Lanka,^10^ likely contributing to primary lung neoplasms being two times more common in males.

By integrating patient demographic characteristics with clinical and radiological features, clinicians can effectively establish precise presumptive diagnoses for lung tumors. This, in turn, facilitates prompt treatment initiation and enhances overall prognosis. Therefore, it is crucial for healthcare professionals to be aware of the regional epidemiological patterns related to the histopathological characteristics of lung tumors. The absence of cancer registry data in Sri Lanka poses a significant constraint, hindering our ability to draw meaningful comparisons with epidemiological findings on a broader scale. Additionally, the unavailability of information on cancer staging and follow‐up survival further curtails the depth of our analysis, emphasizing the importance of future research in these specific areas.

## AUTHOR CONTRIBUTIONS

All authors had full access to the data in the study and take responsibility for the integrity of the data and the accuracy of the data analysis. Conceptualization, I.H. Methodology, I.H., and Yasith Mathangasinghe. Investigation, I.H., Ramani Punchihewa, S.W. and Yasith Mathangasinghe. Formal analysis, Yasith Mathangasinghe and S.W. Resources, Yasith Mathangasinghe. Writing–original draft, Yasith Mathangasinghe. Writing–review and editing, Yasith Mathangasinghe, S.W., I.H., and Ramani Punchihewa.

## CONFLICT OF INTEREST STATEMENT

The authors declare that the research was conducted in the absence of any commercial or financial relationships that could be construed as a potential conflict of interest. This research received no specific grants from any funding agency in the public, commercial or not‐for‐profit sector.

## Data Availability

The original contributions presented in the study are included in the article, further inquiries can be directed to the corresponding author/s.
